# A One-Dimensional Continuum Elastic Model for Membrane-Embedded Gramicidin Dimer Dissociation

**DOI:** 10.1371/journal.pone.0015563

**Published:** 2011-02-04

**Authors:** Joseph N. Stember, Olaf Andersen

**Affiliations:** Department of Physiology and Biophysics, Institute for Computational Biomedicine, Weill Medical College of Cornell University, New York, New York, United States of America; University of Pittsburgh, United States of America

## Abstract

Membrane elastic properties, which are subject to alteration by compounds such as cholesterol, lipid metabolites and other amphiphiles, as well as pharmaceuticals, can have important effects on membrane proteins. A useful tool for measuring some of these effects is the gramicidin A channels, which are formed by transmembrane dimerization of non-conducting subunits that reside in each bilayer leaflet. The length of the conducting channels is less than the bilayer thickness, meaning that channel formation is associated with a local bilayer deformation. Electrophysiological studies have shown that the dimer becomes increasingly destabilized as the hydrophobic mismatch between the channel and the host bilayer increases. That is, the bilayer imposes a disjoining force on the channel, which grows larger with increasing hydrophobic mismatch. The energetic analysis of the channel-bilayer coupling is usually pursued assuming that each subunit, as well as the subunit-subunit interface, is rigid. Here we relax the latter assumption and explore how the bilayer junction responds to changes in this disjoining force using a simple one-dimensional energetic model, which reproduces key features of the bilayer regulation of gramicidin channel lifetimes.

## Introduction

Membrane protein function can be regulated by changes in membrane lipid composition [1]–[8]. This regulation may be due to specific binding to the membrane protein or to changes in bilayer collective properties, such as thickness or lipid intrinsic curvature [9]; the collective properties will be the focus of the present analysis. The latter, physical regulation is important because membrane protein properties change when the membrane lipid composition is altered [9], and because many bioactive molecules are amphiphiles that for thermodynamic reasons [10], [11] will alter lipid bilayer properties, which may provide insight into why amphiphiles modify the function of numerous different membrane proteins [12], [13], [14], [15], (see [16] for a review.) The diversity of membrane proteins that are regulated by a given amphiphile suggests that these compounds may alter membrane protein function by mechanisms that do not involve direct binding to the target protein. In support of this notion, these amphiphiles alter lipid bilayer properties, as sensed by the bilayer-spanning gramicidin channels, at the concentrations where they are promiscuous modulators of membrane protein function. It thus is likely that changes in continuum membrane properties may, quite generally, regulate the function of bilayer-embedded proteins ranging from receptors over channels to transporters and pumps [9]. This is important because drugs – such as genistein [12], capsaicin [13], curcumin [15] and 2,3-butanedione monoxime [17], that may act through specific binding to their target protein over a given concentration range, alter the function of many different membrane proteins at higher concentrations: concentrations at which they modify, to varying degrees, the bulk continuum bilayer properties. These changes in bilayer properties can in turn affect the function of disparate membrane proteins [16], which may lead to undesired side effects [18], [19].

Many different probes have been used to explore how small molecules alter lipid bilayer properties [20], [16]. A particularly useful probe is the gramicidin channel, which is formed from trans-bilayer association of two non-conducting subunits that align “head-to-head” so as to make a continuous, water-filled pore capable of conducting current across the membrane. As such, single-channel measurements can probe the distributions of the monomeric and dimeric states as a function of bulk membrane properties [16]. Specifically, experimental results confirm the hypothesis that the dimer becomes increasingly disfavored with increasing bilayer thickness.

These results usually are interpreted by assuming that the conducting dimer is rigid, compared to the host bilayer, and that there is tight hydrophobic coupling between the membrane-spanning gramicidin dimer and the host bilayer, meaning that the lipid bilayer adjusts to the channel such that the bilayer hydrophobic thickness at the channel-bilayer boundary is equal to the dimer's hydrophobic length. It is further assumed that the channel dissociation occurs as an all-or-nothing phenomenon – meaning that the subunit-subunit interface is assumed to be rigid (until it breaks). While this description is able to account for the observed changes in single-channel lifetime as a function of the channel-bilayer hydrophobic mismatch [21], [16], the subunit-subunit interface is unlikely to be rigid. We therefore expand on the classic description by introducing a simplified one-dimensional description of the bilayer property-dependent channel dissociation with a flexible monomer-monomer interface. The “tug-of-war” between membrane and dimer becomes evident and the resulting changes in dimer lifetime as a function of changes in bilayer thickness reproduce those known from experiment. When we extend the analysis to changes in bilayer stiffness, we again recover the experimentally observed trends. The model is not quantitatively predictive, as it simplifies a complex three-dimensional and many-body problem to a one-dimensional two-body problem. Yet, it illustrates a key phenomenon at play, namely how increasing bilayer thickness affects the joining force between the two subunits, as the bilayer “pulls” the gramicidin dimer apart, and it shows that introducing flexibility at the subunit-subunit interface does not alter the linear relation between channel-bilayer hydrophobic mismatch and the disjoining force acting to pull the dimer apart.

## Methods

### Functional form of the potential and experimental parameters

**Figure 1:**
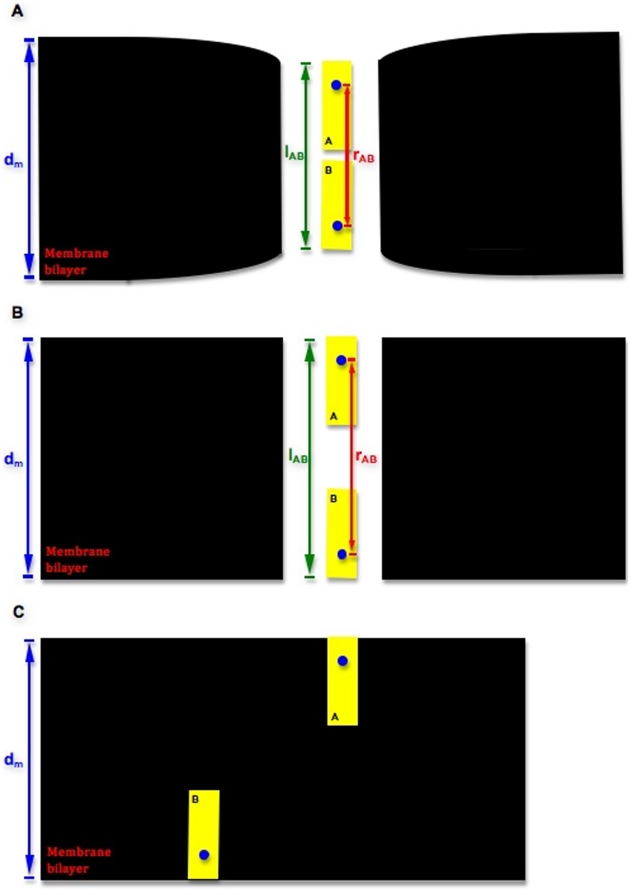
General System Schematic. General schematic of our system. We employ the centers of mass for gramicidin monomers 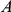 and 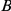 as landmarks to measure monomer separation along the membrane normal axis. The bilayer thickness is denoted by 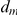, and 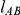 is the hydrophobic length of the gramicidin dimer. The hydrophobic mismatch is given by 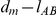. (a) The dimer-state system with equilibrium inter-monomer separation, 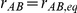. (b) The dissociated state at maximal 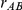 value. (c) The dissociated state in which monomers 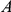 and 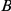 are free to move laterally in the plane of the membrane.

We construct a simple one-dimensional potential to model the gramicidin dimer embedded in a phospholipid bilayer. A general illustration of our simplified system is shown in Figures 1. Figure 1a represents the dimer state at equilibrium. Figure 1b shows the state at which the monomers have separated fully along their common axis. This state gives rise to the dissociated system in Figure 1c, wherein the two monomers have moved apart also in the plane of the bilayer. While the two subunits move apart (or toward each other) axially, The potential consists of a monomer-monomer interaction term 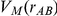 between monomers 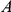 and 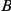, and a membrane-dimer interaction term 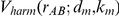, where 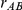 is the distance between 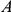 and 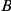
's centers of mass, 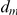 is the membrane thickness and 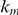 is the membrane stiffness. The monomer-monomer term seeks to restrain 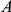 and 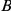 so as to keep 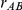 near its equilibrium value of 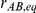. A Morse potential of this form has long been widely used for two-body interaction energies. The membrane-dimer term consists of a harmonic potential that seeks to relieve the hydrophobic mismatch 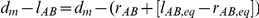 between dimer and membrane, where 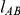 is the dimer hydrophobic length with equilibrium value 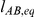 [21]. Our values for 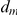 and 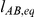 are based on the standard reference model for gramicidin embedded within a membrane [22]: 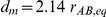 and 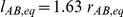.

The full potential is given by
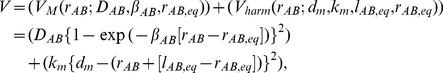
(1)where 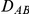 is the dimer dissociation energy and 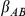 is the corresponding Morse stiffness parameter for the subunit-subunit interaction.

In the conducting state the two monomers are held together by two to six hydrogen bonds [23], [24], though intermediate (presumably low-conductance) states with two and four hydrogen bonds are likely to exist as intermediaries during channel formation and dissociation. The equilibrium distribution of dimers to monomers should be about one to a hundred [25]. We take 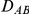 to be 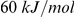. The equilibrium center of mass separation 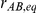 is 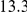 Å. We shall measure all energies in units of 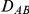 and all distances in units of 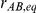. At 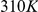, 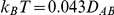. From the atomistic energy profile of Miloshevsky and Jordan [24] with membrane dielectric constant equal to one, the Hookean force constant 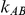 between the monomers can be estimated to be 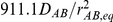. Then the approximation that near the equilibrium distance,
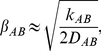
(2)yields 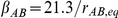. In the theory of elastic bilayer deformations [22], three bilayer material constants (thickness, and the elastic compression and bending moduli) can be combined into a single phenomenological membrane Hookean force coefficient 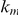. Lundbaek and Andersen [21] estimated 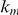 to be 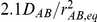 for the gramicidin dimer embedded in a monoglyceride membrane with no solvent.

### Analysis of the potential

**Figure 2:**
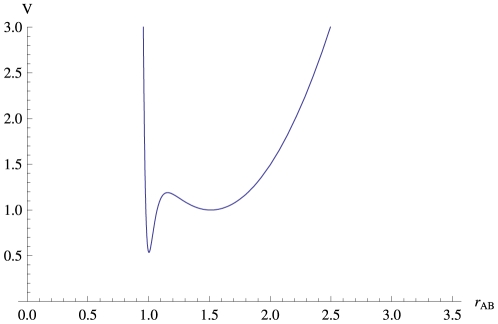
Potential Energy Curve. One-dimensional potential, in units of the dissociation energy 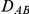, as a function of intermonomer center of mass separation 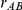, the latter measured in units of equilibrium distance 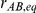.

Figure 2 shows that Equation (1) with this parameter set yields a double-well potential as a function of 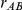. The well on the left (minimum at 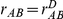) represents the dimer state of gramicidin – corresponding to Figure 1a – and that on the right (minimum at 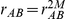) is the separated two-monomer state – corresponding to Figure 1b. A clearly defined transition state (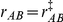) separates the two wells, and we can apply Transition State Theory [26] to express the rate constant for dissociation. Accordingly, we may extract a rate constant 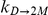 for the dissociation process via the Arrhenius equation:
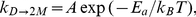
(3)where 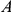 is a pre-exponential factor (the Arrhenius factor), 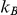 is the Boltzemann constant, 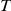 is temperature and 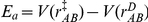 is the activation barrier from the dimer state to the dissociated state. Noting that 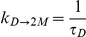, where 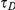 is the average dimer lifetime, we have
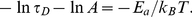
(4)


## Results

**Figure 3:**
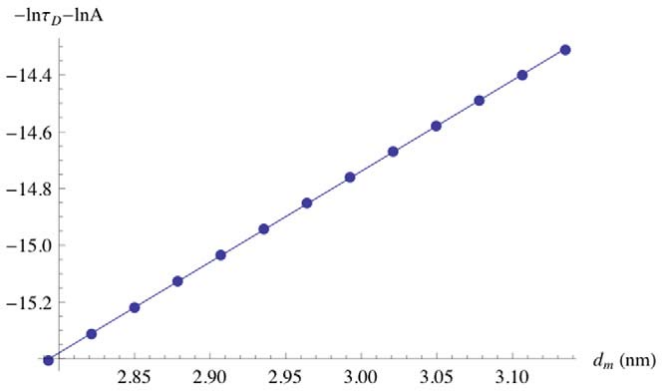
Membrane Thickness Dependence of Dimer Lifetime. The dependence of the dimer lifetime 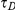 on membrane thickness 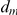, measured in units of nanometers.

**Figure 4:**
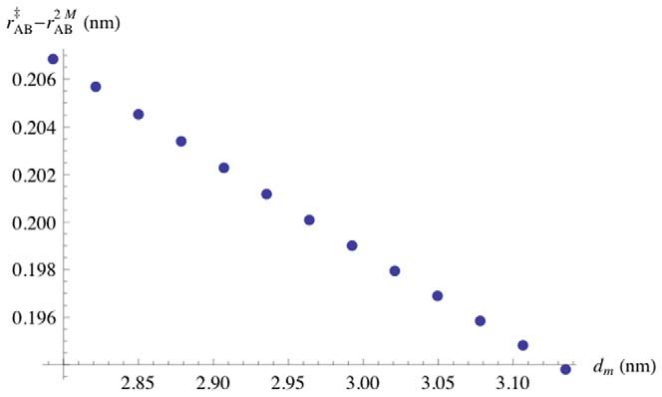
Membrane Thickness Dependence of Distance Between Dimer and Transition State. The distance between dimer and transition state as a function of membrane thickness 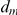, with both quantities being measured in units of nanometers.

As shown previously [21], [16], it is possible to extract the value of a phenomenalogical spring coefficient from the slope of the relation between 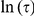 and the channel-bilayer hydrophobic mismatch 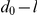, where 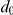 is the bilayer hydrophobic thickness and 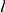 is the channel hydrophobic length – assuming that the distance from the energy minimum for the conducting dimer to the transition state for dimer dissociation does not vary as a function of the hydrophobic mismatch. Given the potential expressed by Equation (1), the relation between 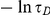 and bilayer thickness remains approximately positive linear (Figure 3), in agreement with experimental results [21], [16]. We therefore examined further how changes in bilayer thickness altered the position of the transition state, i.e. how far the two subunits would move apart in order to reach the transition state, where the channels would stop conducting and move away from each other in the plane of the membrane. The results show that the distance the subunits move apart decreases as the bilayer thickness increases, but that the changes are small, as seen in Figure 4.

**Figure 5:**
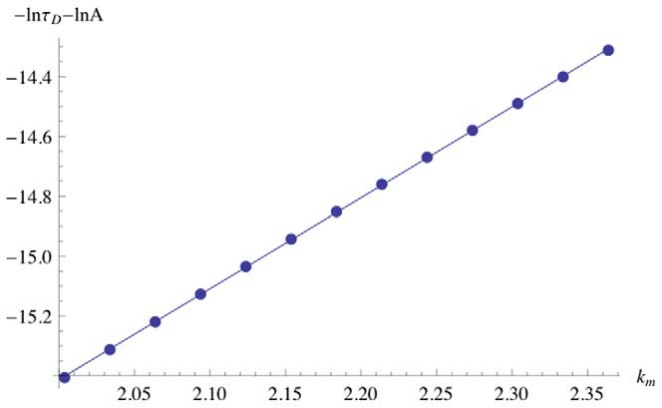
Membrane Stiffness Dependence of Dimer Lifetime. The dependence of the dimer lifetime 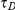 on membrane stiffness 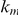, measured in units of 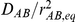.

To examine the experimental trends for varying bilayer stiffness, we scanned through a range of 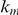 values about the previously noted experimental value while holding membrane thickness 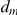 constant. The resulting 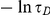 profile, displayed in Figure 5, is an approximately linear relationship similar to that of Figure 3. Though it is not possible to do a quantitative comparison to experimental results, the data reflect the experimental observations [27], [12], [13], [14], [15].

## Discussion

Gramicidin dimer and membrane are engaged in a “tug of war” in which the dimer “wants” to stay close to its equilibrium separation, while the membrane “wants” to pull the monomers apart so as to relieve the bilayer deformation that is caused by the hydrophobic mismatch. The simple two-well potential curve of Figure 2 illustrates two states: the well (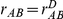) in which the dimer has “won” and stayed in one piece; and the well (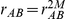) in which the membrane has “won” and successfully pulled the monomers apart to match up the channel's hydrophobic length with the membrane thickness. The actual dissociation is, of course, more complex than depicted here, most likely being a coupled separation along the bilayer normal and rotation/translation in the plan of the bilayer [28]. The key point here is that the monomers separate along their major axes (i.e. 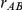 increases), then at some point fully break apart, and finally drift apart in the plane of the membrane (Figure 1c).

Subject to this limitation, our model demonstrates that the essential features of the dimer dissociation remain intact as we vary bilayer thickness. Doing so, the distance to the transition state decreases as the bilayer thickness increases (as the disjoining force increases), but the relative change in this distance is modest, indicating that it is indeed possible to use the lifetime-thickness relation, as determined using gramicidin channels, to estimate changes in bilayer properties. A more detailed analysis, with coarse-grained or atomistic simulation, is needed to thoroughly understand the mechanism of gramicidin separation, this process's dependence on the membrane's chemistry and bulk properties, and the limitations encountered in the experiments.
